# Sensory neuropathy hampers nociception-mediated bone marrow stem cell release in mice and patients with diabetes

**DOI:** 10.1007/s00125-015-3735-0

**Published:** 2015-09-10

**Authors:** Zexu Dang, Davide Maselli, Gaia Spinetti, Elena Sangalli, Franco Carnelli, Francesco Rosa, Elena Seganfreddo, Fabio Canal, Anna Furlan, Agostino Paccagnella, Emanuela Paiola, Bruno Lorusso, Claudia Specchia, Mattia Albiero, Roberta Cappellari, Angelo Avogaro, Angela Falco, Federico Quaini, Kepeng Ou, Iker Rodriguez-Arabaolaza, Costanza Emanueli, Maria Sambataro, Gian Paolo Fadini, Paolo Madeddu

**Affiliations:** Bristol Heart Institute, School of Clinical Sciences, University of Bristol, Upper Maudlin Street, Bristol, BS2 8HW UK; I.R.C.C.S. (Scientific Institute of Medical Research) MultiMedica, Milan, Italy; Department of Pathology, Santa Maria of Ca’ Foncello Hospital, Treviso, Italy; Department of Specialized Medicines, Hematology Unit, Santa Maria of Ca’ Foncello Hospital, Treviso, Italy; Department of Specialized Medicines, Endocrine, Metabolic and Nutrition Diseases Unit, Santa Maria of Ca’ Foncello Hospital, 1 Piazza Ospedale, 31100 Treviso, Italy; Clinical and Experimental Medicine, University of Parma, Parma, Italy; Department of Molecular and Translational Medicine, University of Brescia, Brescia, Italy; Department of Medicine, University of Padova, Padova, Italy

**Keywords:** Bone marrow, Diabetes mellitus, Nociception

## Abstract

**Aims/hypothesis:**

Upon tissue injury, peripheral sensory neurons release nociceptive factors (e.g. substance P [SP]), which exert local and systemic actions including the recruitment of bone marrow (BM)-derived haematopoietic stem and progenitor cells (HSPCs) endowed with paracrine pro-angiogenic properties. We herein explore whether diabetic neuropathy interferes with these phenomena.

**Methods:**

We first investigated the presence of sensory neuropathy in the BM of patients with type 2 diabetes by immunohistochemistry and morphometry analyses of nerve size and density and assessment of SP release by ELISA. We next analysed the association of sensory neuropathy with altered HSPC release under ischaemia or following direct stimulation with granulocyte colony-stimulating factor (G-CSF). BM and circulating HSPCs expressing the neurokinin 1 receptor (NK1R), which is the main SP receptor, were measured by flow cytometry. We finally assessed whether an altered modulation of SP secretion interferes with the mobilisation and homing of NK1R-HSPCs in a mouse model of type 2 diabetes after limb ischaemia (LI).

**Results:**

Nociceptive fibres were reduced in the BM of patients and mice with type 2 diabetes. Patients with neuropathy showed a remarkable reduction in NK1R-HSPC mobilisation under ischaemia or upon G-CSF stimulation. Following LI, diabetic mice manifested an altered SP gradient between BM, peripheral blood and limb muscles, accompanied by a depressed recruitment of NK1R-HSPCs to the ischaemic site.

**Conclusions/interpretation:**

Sensory neuropathy translates into defective liberation and homing of reparative HSPCs. Nociceptors may represent a new target for treatment of diabetic complications.

**Electronic supplementary material:**

The online version of this article (doi:10.1007/s00125-015-3735-0) contains peer-reviewed but unedited supplementary material, which is available to authorised users.

## Introduction

Diabetic patients manifest a defective release of haematopoietic stem and progenitor cells (HSPCs) following tissue injury, ischaemia or stimulation by granulocyte colony-stimulating factor (G-CSF), a condition referred to as diabetic stem cell mobilopathy [1]. This feature is emerging as a clinically relevant pathology associated with increased risk of vascular complications [1, 2]. Structural and functional abnormalities of the bone marrow (BM) microenvironment might contribute to these phenomena [3]. A better mechanistic understanding might help in the introduction of new strategies to counteract the consequences of mobilopathy in diabetic patients.

New exciting evidence indicates that an intact nociceptive system is crucial for the recruitment of regenerative cells from the circulation to ischaemic tissues. We have recently reported that, in otherwise healthy mice, ischaemia triggers differential nociceptor responses in peripheral tissues and BM, thereby generating a gradient of neuropeptide substance P (SP)/neurokinin 1 that favours the release and homing of HSPCs expressing the NK1 receptor (NK1R), which is the main receptor for SP [4]. Recruitment of NK1R-HSPCs to the ischaemic site helps tissue healing through paracrine stimulation of reparative angiogenesis. The crucial contribution of this mechanism to post-ischaemic recovery is highlighted by the observation that BM reconstitution with *Nk1r*-knockout (also known as *Tacr1*-knockout) HSPCs results in depressed HSPC mobilisation, delayed blood-flow recovery and reduced neovascularisation after ischaemia. [4] Importantly, this mechanism participates in modulation of the HSPCs’ mobilisation in humans. In fact, patients with acute myocardial infarction show high circulating and cardiac levels of SP and NK1R-HSPCs whereas these phenomena are abrogated in infarcted patients with a denervated transplanted heart [4].

Diabetic neuropathy is a common and disabling manifestation of diabetes mellitus and often overlaps with vascular complications [5, 6]. Direct neuronal damage caused by hyperglycaemia and downregulation of neurotrophic factors concurs in the pathogenesis of diabetic neuropathy [7, 8]. Sensory neuropathy is a typical form of peripheral neuropathy characterised by an altered perception of noxious stimuli [9–12]. From a functional standpoint, it manifests as an inability of neurons to produce proper amounts of neuropeptides, like SP and calcitonin-gene related peptide, in response to tissue injury. Altered nociception facilitates foot ulcers caused by pressure or trauma and abrogates warning symptoms during a heart attack. These consequences are mainly attributed to failure of peripheral healing mechanisms. It is not yet known whether extension of sensory neuropathy to the BM may contribute to altered stem cell release. Additionally, direct stimulation of BM-HSPC liberation by G-CSF is frequently associated with intense bone pain. Although peripheral sympathetic nerves reportedly participate in G-CSF-induced mobilisation [13, 14], the contribution of a dysfunctional nociceptive signalling in diabetic BM unresponsiveness to G-CSF has not been addressed so far.

This study investigates whether sensory neuropathy extends to the BM of patients and mice with diabetes and plays a role in stem cell mobilopathy. Results indicate for the first time that diabetic sensory neuropathy contributes towards hindrance of cellular mechanisms of tissue repair.

## Methods

Details of methodology and inclusion/exclusion criteria are provided in the electronic supplementary material (ESM) Methods.

### Human studies

We conducted four distinct studies to verify the presence of sensory neuropathy in BM and its association with defective HSPC release under ischaemia or following direct stimulation.

We carried out two cross-sectional studies. The first was an observational pathology study assessing the presence of neuropathy in the BM of patients with type 2 diabetes (either uncomplicated or complicated with foot lesions) and its association with alteration in the abundance of antigenically defined HSPC subpopulations. Consecutive patients with type 2 diabetes and non-diabetic controls were recruited at Santa Maria di Ca’ Foncello Hospital (Treviso), IRCCS MultiMedica (Milan) and the University Hospital of Parma, under Ethics Committee approval and after obtaining informed consent (ESM Tables 1 and 2). Non-diabetic individuals were enrolled among those referred for hip replacement surgery or diagnostic BM examination to exclude a haematological disease. The patients with type 2 diabetes were subdivided into three groups: (1) uncomplicated (T2DM-U), (2) with peripheral neuropathy (T2DM-N) and (3) with peripheral neuropathy and critical limb ischaemia (CLI) (T2DM-NI). Peripheral neuropathy was defined using the diabetic neurological index and neuropathy disability score [9, 15] and autonomic neuropathy using the criteria of the American Autonomic Society and the American Academy of Neurology [16, 17]. CLI was diagnosed based on TASC 2007 criteria [18].

The second cross-sectional study was aimed at confirming the alteration of SP receptor NK1R expression on circulating CD34^+^ HSPCs in an independent cohort of patients with type 2 diabetes referred to the Outpatient Clinic of the University Hospital of Padova, under approval of the local ethics committee and after obtaining informed consent (ESM Table 3).

In addition, two intervention studies retrospectively verified the relationship between HSPCs mobilisation and nociception. First, we re-analysed the results of a trial conducted in 40 healthy volunteers, in which the primary endpoint was to determine the effect of subcutaneous pegylated human recombinant G-CSF on the mobilisation of HSPCs [19]. Back pain and bone pain were given a score of 1, 2 or 3 for mild, moderate or severe intensity, respectively. Duration of pain in days was recorded. A final pain score was calculated as intensity × duration. Patients were then divided into two equal groups according to the median value of their pain score and the absolute average change in CD34^+^ cells/ml before and after G-CSF was calculated for each group. The placebo group was used as a control for pain and change in CD34^+^ cell counts.

Finally, we re-analysed the results of a recent trial performed at the University Hospital of Padova, the primary endpoint of which was to compare the CD34^+^ cell mobilisation response to low-dose human recombinant G-CSF in non-diabetic individuals and diabetic patients (NCT01102699) [2, 20]. Changes in peripheral blood (PB) CD34^+^ cell count and SP levels from baseline were computed according to the presence or absence of G-CSF-induced pain (ESM Table 4).

### Animal studies

Experiments were performed in accordance with the Guide for the Care and Use of Laboratory Animals (The Institute of Laboratory Animal Resources, 1996) and with approval of the University of Bristol and the British Home Office (Licence: 30/2811). As a model of type 2 diabetes, we used 16-week-old male obese leptin-receptor homozygous mutant BKS.Cg-*Lepr*^db^/*Lepr*^db^/OlaHsd (*db/db*) mice (Harlan, Blackthorn, UK). Elevation of blood glucose starts at 4–8 weeks of age, followed by manifestation of neuropathy [21]. Age- and sex-matched lean (BKS.Cg-*m*^+/+^*Lepr*^db^/OlaHsd) (*db/+*) mice served as controls.

As a model of reparative neovascularisation, we applied unilateral limb ischaemia (LI), induced by ligature of the left femoral artery [22]. Mice were killed at 0, 3, 7, 14 or 21 days after LI (*n* = 5 mice per group) and PB and BM from tibias and femurs were collected for analyses.

### Analytical measurements

ELISA kits were used to determine SP levels (Cayman Chemical, Ann Arbor, Michigan, USA).

For flow cytometry experiments, whole blood was collected in tubes containing EDTA. BM cells were obtained by flushing mouse femoral marrow. In human studies, marrow was either sampled from the posterior iliac crest or collected from bone remnants of hip surgery. Cells were also isolated by enzymatic digestion from the adductor muscles of mice undergoing LI. Quantification of antigenically defined cell populations was conducted by flow cytometry (concentration of antibodies is shown in ESM Table 5).

In the immunohistochemistry and immunofluorescence studies, decalcified BM biopsies were embedded in paraffin and sectioned at 2 μm. Sections were then incubated with primary and secondary antibodies (ESM Table 5) following standard procedures.

### Statistical analysis

Continuous variables were compared among groups using ANOVA, with post hoc Student’s *t* test after Bonferroni correction. Categorical variables were compared using the *χ*^2^ test. The association between continuous variables was evaluated using Pearson’s *r* coefficient. The *p* values are two-sided and statistically significant when <0.05.

Multiple linear regression analyses were used to adjust for clinical and demographical variables that resulted in an imbalance between groups. Analyses were performed with Stata 12 (Stata Statistical Software, College Station, TX, USA) Graphs were generated using GraphPad Prism 5 (GraphPad Software, La Jolla, CA, USA).

## Results

### Pathology study showing the association of sensory neuropathy and stem cell mobilopathy in patients with type 2 diabetes

**Fig. 1:**
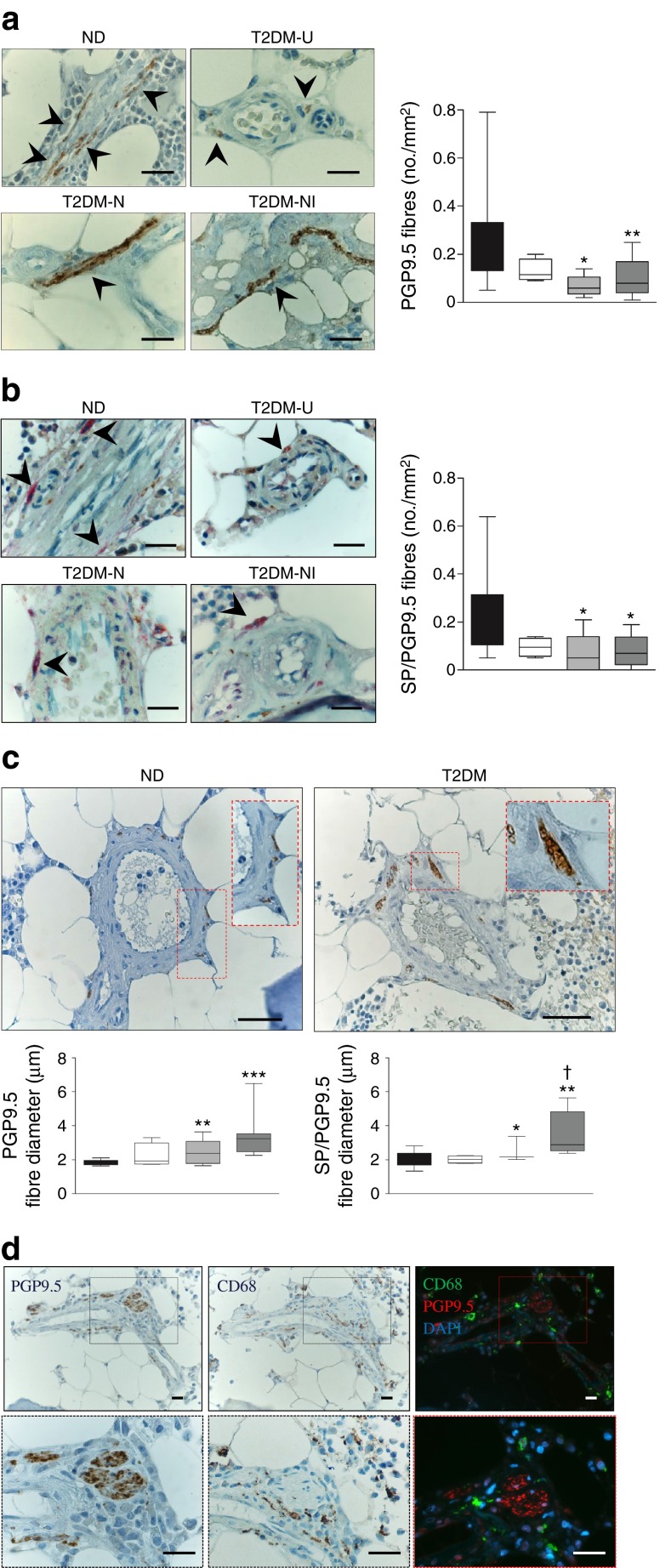
Rarefaction and degeneration of sensory fibres in the BM of patients with type 2 diabetes. (**a**) Representative micrographs and quantification of neuronal fibres expressing the pan-neuronal marker PGP9.5 (brown, indicated by arrowheads) (scale bar, 20 μm). Box and whisker graphs illustrate the difference between non-diabetic individuals (ND, black box, *n* = 18) and patients with type 2 diabetes (*n* = 24) classified as T2DM-U (white box, *n* = 4), T2DM-N (light grey box, *n* = 5) or T2DM-NI (dark grey box, *n* = 15). *p < 0.05 and **p < 0.01 vs ND. (**b**) Representative micrographs and quantification of neuronal fibres co-expressing PGP9.5 (brown) and SP (red) (scale bar, 20 μm). Box and whisker graphs illustrate the difference between groups. *p < 0.05 vs ND. (**c**) Micrographs represent typical neuronal fibre features in non-diabetic individuals and patients with type 2 diabetes mellitus (T2DM) (scale bar, 50 μm). Box and whisker graphs illustrate neuronal fibre diameter in study groups. *p < 0.05, **p < 0.01 and ***p < 0.001 vs ND and ^†^ *p* < 0.05 vs T2DM-U. (**d**) Typical immunohistochemistry and immunofluorescence staining of subsequent BM slides from the same patient with type 2 diabetes shows infiltration of the perineurium by CD68^+^ macrophages. Lower panels are magnifications of the fields delimited by red dotted squares, (scale bar, 20 μm)

**Fig. 2:**
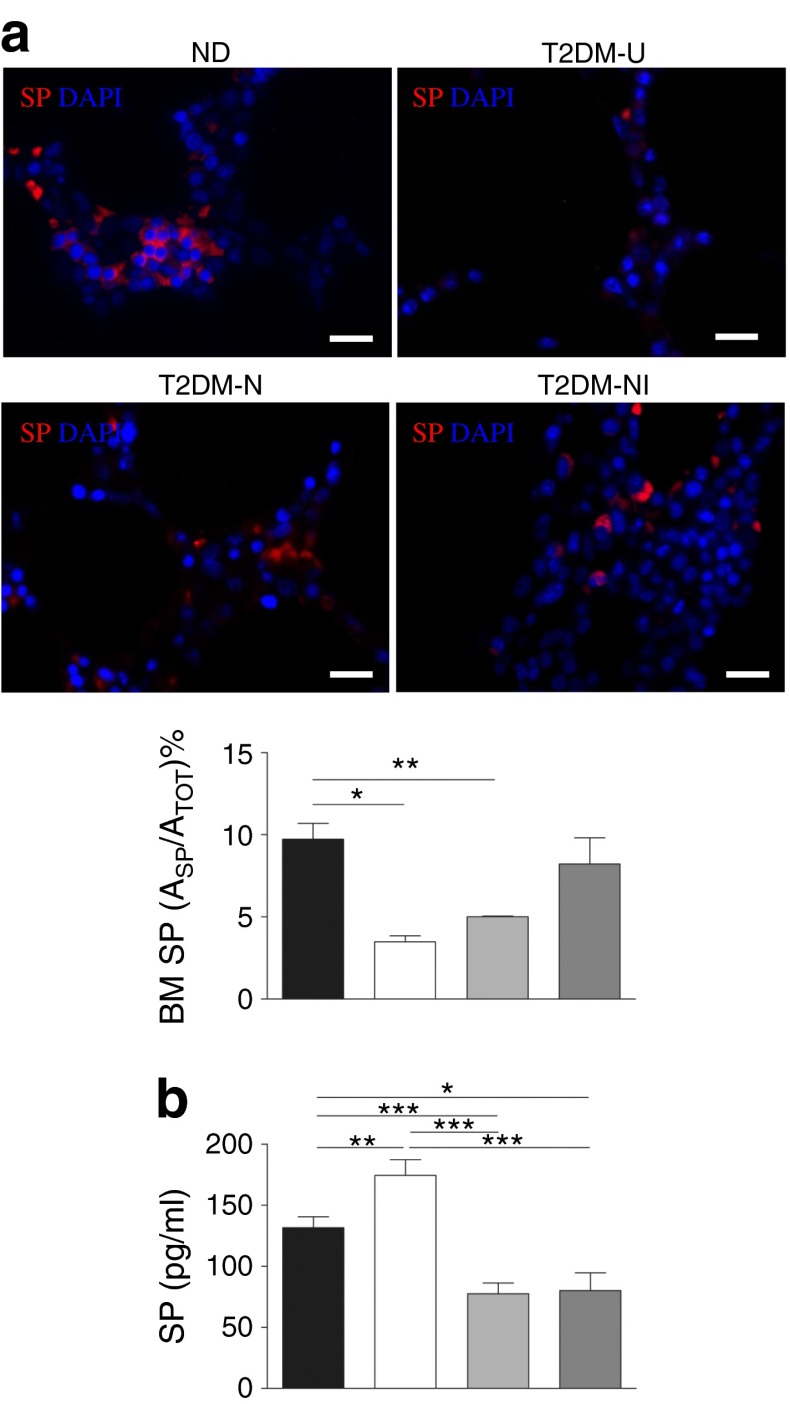
Type 2 diabetes alters the levels of SP in PB and BM. (**a**) Immunostaining for SP in BM and graph showing SP levels in the BM of non-diabetic individuals; positive SP-stained area (A_SP_) as a percentage of the total area of sections (A_TOT_). (ND, *n* = 7) and patients with type 2 diabetes (*n* = 4, each subgroup [see (**b**)]) (scale bar, 50 μm). (**b**) Bar graphs (mean and SEM) showing quantitative analysis of immunoreactive SP in the PB of non-diabetic controls (black bar, *n* = 15) and patients with type 2 diabetes classified as uncomplicated (T2DM-U, white bar, *n* = 13) or complicated by neuropathy (T2DM-N, light grey bar, *n* = 35) or neuropathy and CLI (T2DM-NI, dark grey bar, *n* = 33). *p < 0.05, **p < 0.01 and ***p < 0.001 for indicated comparisons

We first sought evidence of sensory neuropathy in the BM of patients with type 2 diabetes by conducting an analysis of neuronal fibre density on specimens collected from iliac crest biopsies or bone left over from hip reconstructive surgery. Since nerve density was similar in the two source groups, data were pooled in the final analysis. By immunohistochemistry, we recognised a striking decrease in the total number of nerve fibres that express the general neuronal marker PGP9.5 in the BM of diabetic patients with neuropathic (T2DM-N) and neuropathic/ischaemic complications (T2DM-NI) (Fig. 1a, ANOVA *p* < 0.001). This defect extends to nociceptive fibres that co-express PGP9.5 and SP (Fig. 1b, ANOVA *p* < 0.01) and to tyrosine hydroxylase-positive sympathetic fibres (ESM Fig. 1a, *p* < 0.05 vs non-diabetic controls). Additionally, in the BM of T2DM-N and T2DM-NI patients, both PGP9.5-positive and SP-positive nerve fibres showed a marked increase in diameter (Fig. 1c, ANOVA *p* < 0.0001 and *p* < 0.01, respectively), as well as vacuolisation and infiltration with CD68-positive macrophages (Fig. 1d). These degenerative/inflammatory features were not observed in non-diabetic controls. This association of diabetes with nociceptive fibre rarefaction and remodelling was additionally confirmed in a multivariable analysis adjusted by age, BMI, fasting glucose and HbA_1c_. Furthermore, neuronal rarefaction was associated with reduction in BM capillary density (ESM Fig. 1b), which identifies a distinct form of microangiopathy we have described previously in animal models and patients with diabetes [23, 24]. Furthermore, by immunofluorescence microscopy, we detected a significant reduction in SP expression in the BM of diabetic patients with neuropathy (*p* < 0.01 vs non-diabetic controls) but not in those with superimposed CLI (Fig. 2a). In addition, SP levels were remarkably reduced in the PB of neuropathic diabetic patients with or without CLI (*p* < 0.001 vs non-diabetic controls; *p* < 0.001 vs uncomplicated diabetes) (Fig. 2b).

**Fig. 3:**
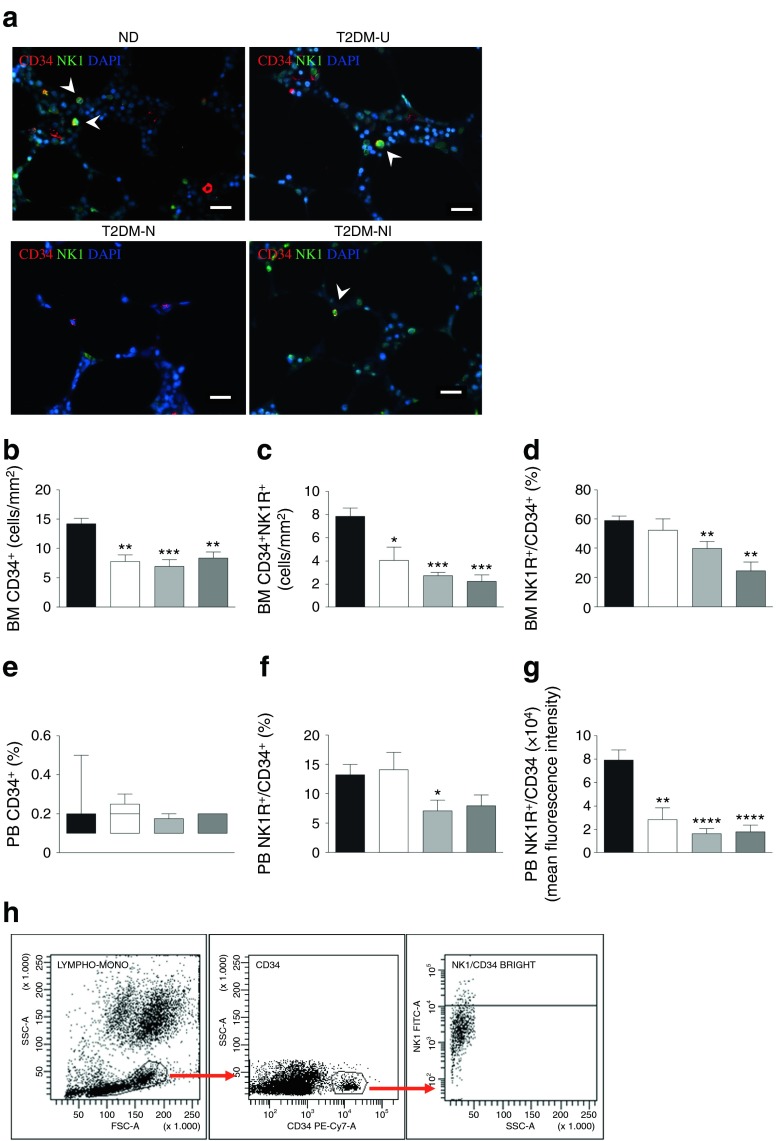
Impaired release of CD34^+^NK1R^+^ cells in patients with type 2 diabetes and neurovascular complications. (**a**) Immunofluorescence micrograph of BM CD34^+^NK1R^+^ cells (scale bar, 20 μm). ND, non-diabetic. Arrowheads show cells positive for CD34 and NK1R markers. (**b**–**d**) Bar graphs showing the levels of CD34^+^ (**b**) and CD34^+^NK1R^+^ cells (**c**) and relative abundance of NK1R^+^ cells over total CD34^+^ cells (**d**). Black bars, non-diabetic (*n* = 6); white bars, T2DM-U (*n* = 3); light grey bars, T2DM-N (*n* = 5); dark grey, T2DM-NI (*n* = 10). (**e**–**g**) Bar graphs showing the abundance of CD34^+^ (**e**) and NK1R^+^ cells on total CD34^+^ cells (**f**) (*p* = 0.06, non-diabetic vs T2DM-NI) in the PB of non-diabetic individuals and patients with type 2 diabetes, assessed by flow cytometry (non-diabetic, *n* = 8; T2DM-U, *n* = 5; T2DM-N, *n* = 12; T2DM-NI, *n* = 11); the NK1R mean fluorescence intensity on CD34^+^ cells is shown in (**g**). (**h**) Dot plots of gating strategy. *p < 0.05, **p < 0.01, ***p < 0.001 and ****p < 0.0001 vs non-diabetic controls

Having documented the presence of structural and functional nociceptive alterations in the BM of patients with type 2 diabetes, we next conducted immunofluorescence and flow cytometry analyses of CD34^+^ HSPCs, which co-express the SP receptor NK1R. Patients with complicated diabetes showed a depletion of CD34^+^NK1R^+^ HSPCs in their BM (Fig. 3a–d) and PB (Fig. 3e–h). Mean fluorescence intensity analysis indicated that NK1R was downregulated in cells from diabetic patients (Fig. 3g). Furthermore, superimposed CLI failed to induce an increment in circulating CD34^+^NK1R^+^ HSPCs (Fig. 3e–h). Altogether, these data indicate that sensory neuropathy is associated with BM depletion and altered release of a subpopulation of HSPCs that is responsive to SP chemoattraction. In particular, the presence of ischaemia, which reportedly acts as a potent stimulus for CD34^+^NK1R^+^ HSPC release [4], fails to promote cell mobilisation in patients with type 2 diabetes.

To confirm the presence of a specific form of mobilopathy affecting the subpopulation of CD34^+^NK1R^+^ HSPCs, we investigated an independent cohort of 24 non-diabetic individuals and 74 patients with type 2 diabetes (ESM Table 3 and ESM Fig. 2). A multivariable analysis showed reduced PB levels of CD34^+^NK1R^+^ HSPCs in the diabetic patients as compared with non-diabetic individuals, even after adjustment for BMI and medications that were significantly different at univariate analysis.

### Experimental study showing the inhibitory effect of type 2 diabetes on nociceptive-mediated HSPC mobilisation and homing in mice with induced limb ischaemia

**Fig. 4:**
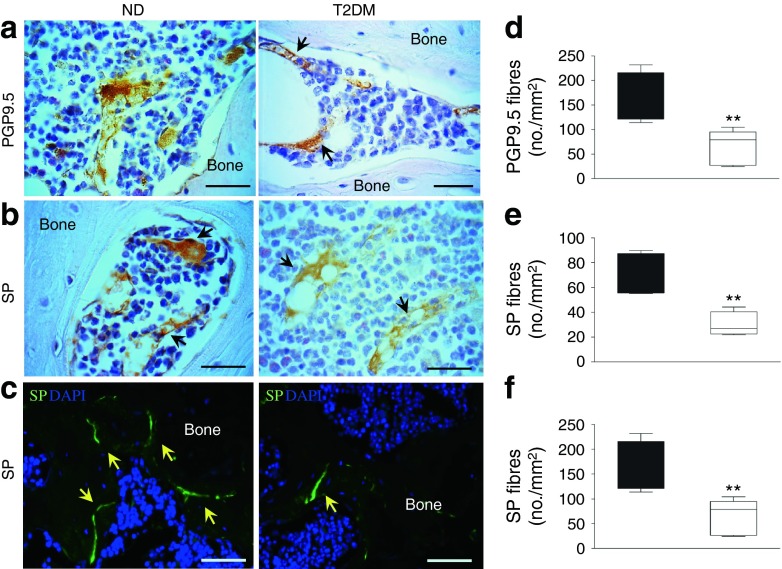
Rarefaction of sensory fibres in the BM of diabetic mice. Immunohistochemistry studies were carried out to compare the nerve fibre density in the BM of mice with type 2 diabetes (T2DM) and respective non-diabetic controls (ND). (**a**, **b**) Representative micrographs showing the density of neuronal fibres expressing the pan-neuronal marker PGP9.5 (**a**) and nociceptive fibres positive for SP (**b**) (scale bar, 20 μm). Arrows point to positive fibres. (**c**) Representative immunofluorescence microscopy images of SP-positive fibres (arrows) (scale bar, 50 μm). (**d**–**f**) Box and whisker graphs show morphometry data. Boxes (black, non-diabetic; white, type 2 diabetes) are bordered at the 5th and 95th percentiles of the variable with a median line at the 50th percentile. Whiskers extend from the box to the upper and lower adjacent values and are capped with an adjacent line. **p < 0.01 vs non-diabetic control; *n* = 5 per group

We next conducted studies to compare the abundance of nerve fibres in the BM of *Lepr*^db/db^ diabetic mice and non-diabetic controls and to investigate the effect of experimental type 2 diabetes on NK1R^+^ HSPC liberation as stimulated by peripheral ischaemia. The abundance of neuronal fibres expressing the pan-neuronal marker PGP9.5 and nociceptive SP-expressing nerves was measured in the marrow (Fig. 4a, b) and compact bone (Fig. 4c). Morphometric analyses indicated that diabetes induces a marked reduction in the density of both PGP9.5-positive fibres (Fig. 4d, *p* < 0.01 vs non-diabetic control) and SP-containing sensory terminals (Fig. 4e, f, *p* < 0.01 vs non-diabetic control).

**Fig. 5:**
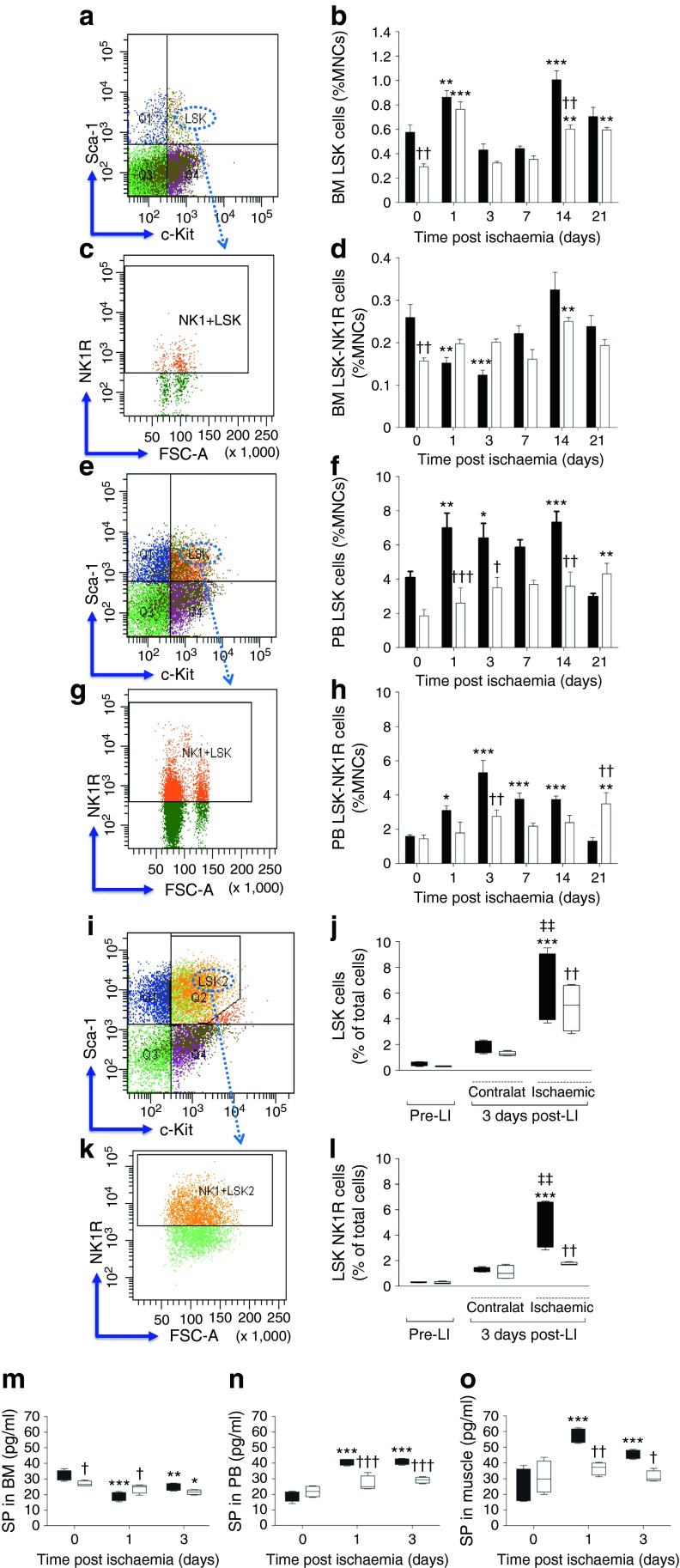
Impairment of the nociceptive mechanism is associated with reduced liberation and homing of stem cells in diabetic mice submitted to unilateral LI. (**a**–**h**) Flow cytometry analyses showing the abundance of LSK and LSK-NK1R cells in the BM (**a**–**d**) and PB (**e**–**h**) of diabetic (white bars) and non-diabetic (black bars) mice, before and after induction of unilateral LI. Data expressed as percentage of mononuclear cells (MNCs) *p < 0.05, **p < 0.01 and ***p < 0.001 vs time 0; ^††^ *p* < 0.01 and ^†††^ *p* < 0.001 vs non-diabetic control; *n* = 5 per group. (**i**–**l**) Flow cytometry analyses showing the abundance of LSK (**i**, **j**) and LSK-NK1R (**k**, **l**) cells in ischaemic and contralateral limb muscles of non-diabetic (black boxes) and diabetic (white boxes) mice at 3 days post-LI compared with pre-LI. ***p < 0.001 vs pre-LI; ^††^ *p* < 0.01 vs non-diabetic control; ^‡‡^ *p* < 0.01 vs contralateral; *n* = 5 per group. The gating strategy is shown in (**i**) and (**k**). (**m**–**o**) Levels of SP in the BM (**m**), PB (**n**) and ischaemic muscles (**o**) of non-diabetic (black boxes) and diabetic (white boxes) mice before and after induction of LI. *p < 0.05, **p < 0.01 and ***p < 0.001 vs pre-LI; ^†^ *p* < 0.05, ^††^ *p* < 0.01 and ^†††^ *p* < 0.001 vs non-diabetic control; *n* = 5 per group

Remarkable differences were observed in the tissue redistribution of HSPC populations following LI in non-diabetic mice compared with diabetic mice. In both groups, LI caused an initial expansion of LSK-HSPCs in the BM, and this was followed by a return to basal levels at 3–7 days post-LI and then a second peak at 14 days (Fig. 5a, b). The subpopulation of LSK-NK1R-HSPCs was reduced in the BM of non-diabetic mice during the acute phase of LI, returning to basal levels by day 7. In contrast, no change was observed in diabetic mice before and after LI (Fig. 5c, d). When looking at cellular changes in PB, we observed a striking increase in LSK-HSPCs and LSK-NK1R-HSPCs from day 1 to day 14 post-LI in non-diabetic mice, with this response being remarkably attenuated and delayed in diabetic mice (Fig. 5e–h, *p* < 0.01 vs non-diabetic mice). Additionally, the diabetic mice manifested a reduction in the homing of LSK-HSPCs and LSK-NK1R-HSPCs at the level of the ischaemic limb muscle, whereas no difference between groups was seen in the contralateral muscle (Fig. 5i–l, *p* < 0.01 vs non-diabetic mice).

We next investigated whether these deficits associate with alterations of nociceptor-related mechanisms. To this aim, we measured the levels of SP in BM supernatant fractions, PB and ischaemic muscles of diabetic and non-diabetic mice before and after LI. Two-way ANOVA detected an effect of time (*p* < 0.01) and group factor (*p* < 0.01) on the levels of SP in these different compartments; specifically, non-diabetic mice manifested a positive gradient of SP between muscle, PB and BM following LI, whereas this phenomenon was much attenuated in diabetic mice (Fig. 5m–o and ESM Fig. 3).

Since recruitment of LSK-NK1R-HSPCs has been shown to be essential for post-ischaemic vasculogenesis [4], we anticipated that the observed cellular defect may participate in depressing the spontaneous recovery from ischaemia. Consistently, the diabetic mice showed remarkable deficits in limb blood-flow recovery (ESM Fig. 4a, b, *p* < 0.01) and reparative capillary angiogenesis (ESM Fig. 4c, d, *p* < 0.001) when compared with non-diabetic mice.

### Clinical studies showing that dysfunctional nociception contributes to defective G-CSF-induced mobilisation

Having shown that diabetes-induced nociceptive dysfunction contributes to depressed HSPC release in ischaemia, we next investigated the relation between pain, circulating levels of SP and CD34^+^ HSPC mobilisation following direct stimulation of BM with G-CSF.

**Fig. 6:**
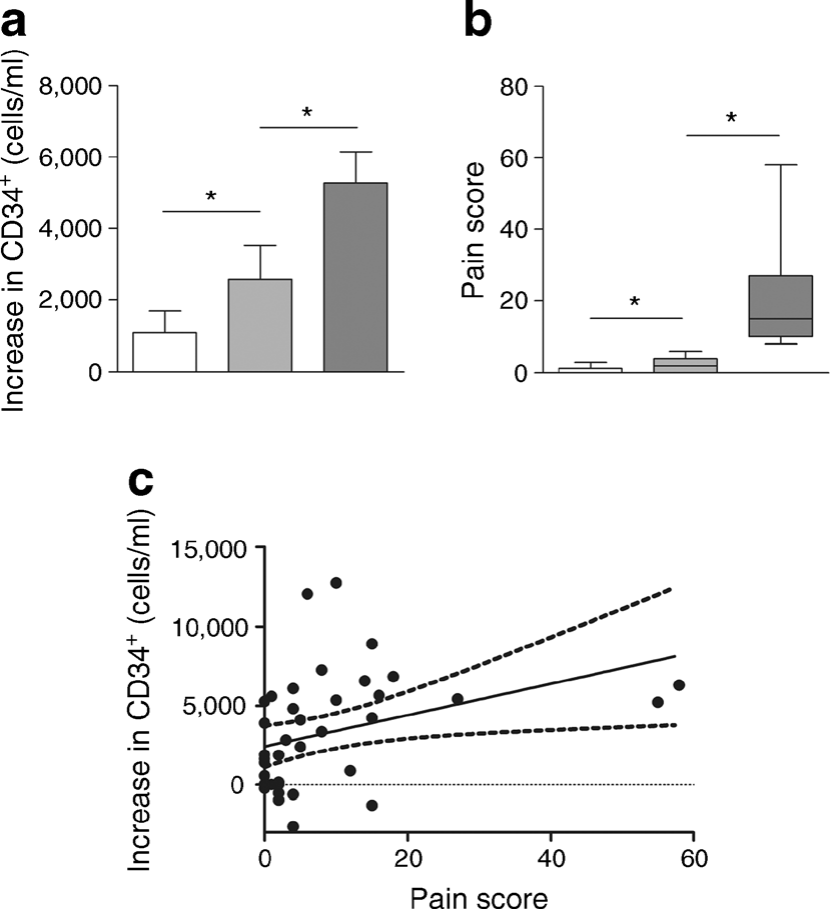
CD34^+^ cell mobilisation induced by G-CSF administration correlates with pain score in non-diabetic individuals. (**a**) Changes (mean ± SEM) in PB CD34^+^ cells of individuals given placebo (white bar, *n* = 10) or given G-CSF and classified as reporting no/mild bone/back pain (light grey bar, *n* = 15) or moderate/severe pain (dark grey bar, *n* = 15); *p < 0.05 for indicated comparison. (**b**) A pain score was computed as described in the Methods section and plotted; *p < 0.05 for indicated comparison. (**c**) Linear correlation between the pain score and the degree of CD34^+^ cell mobilisation in individuals who received G-CSF (*r* = 0.36; *p* = 0.016); dashed lines represent the 95% CI and dotted line shows the grid line at *y* = 0

A cohort of healthy volunteers received placebo or recombinant human G-CSF. Those receiving G-CSF were grouped according to the level of pain (graded as pain score) induced by the growth factor. Interestingly, individuals who experienced moderate to severe bone or back pain showed a significantly greater increase in PB CD34^+^ HSPC counts than those who reported no pain or mild pain (Fig. 6a, b). There was a significant direct correlation between the pain score and the increase in the number of CD34^+^ HSPCs induced by G-CSF (*r* = 0.36, *p* < 0.02; Fig. 6c).

**Fig. 7:**
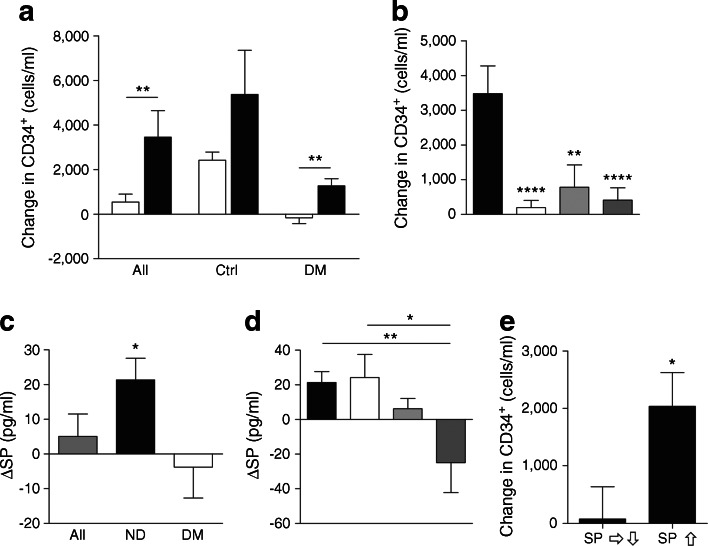
Complicated diabetes inhibits CD34^+^ cell mobilisation induced by G-CSF. (**a**) Changes (mean ± SEM) in the PB levels of CD34^+^ cells in patients grouped according to the presence (DM) or absence (Ctrl) of diabetes and the presence (black bars) or absence (white bars) of pain after G-CSF stimulation. **p < 0.01, pain vs no pain; in non-diabetic individuals *p* = 0.07 for pain vs no pain. (**b**) Changes in the PB levels of CD34^+^ cells before and after G-CSF stimulation in patients grouped according to diabetes complications (white bar, no complications; light grey bar, neuropathy; dark grey bar neuroischaemia). **p < 0.01 and ****p < 0.0001, vs non-diabetic (black bar). (**c**) Changes in SP concentrations induced by G-CSF stimulation in diabetic (DM) patients and non-diabetic individuals (ND). *p < 0.05 ND vs DM. (**d**) Changes in SP concentrations induced by G-CSF stimulation in diabetic patients grouped according to complications (see [**b**] for key). *p < 0.05 and **p < 0.01, for indicated comparisons. (**e**) Changes in the levels of CD34^+^ cells after G-CSF stimulation in individuals showing stable or reduced SP levels vs those showing an increase in SP levels (*p < 0.05)

We also re-analysed data from a trial of BM stimulation with human recombinant G-CSF in diabetic and non-diabetic individuals. Specifically, here we investigated the association of HSPC mobilisation and pain perception. In the whole cohort, the individuals who reported back or bone pain after G-CSF administration showed a significantly higher increase in PB CD34^+^ HSPCs than those reporting no pain (*p* < 0.01, Fig. 7a). Two-way ANOVA detected an inhibitory effect of diabetes (*p* < 0.0001) and an enhancing effect of pain (*p* < 0.01) on G-CSF-induced mobilisation, with no interaction between factors. Furthermore, in diabetic patients, mobilisation was completely abrogated in the absence of pain (Fig. 7a) and there was no incremental effect of vascular complications on mobilisation in comparison with diabetic patients without vascular disease (Fig. 7b). To determine whether depressed nociceptive signals may account for the reduced mobilisation of HSPCs in diabetic patients, we measured PB SP concentrations before and 24 h after G-CSF injection. Interestingly, G-CSF administration caused an increase in SP levels in non-diabetic individuals (*p* < 0.05), with this response being abrogated in diabetic patients (Fig. 7c). Additionally, in diabetic patients with neuropathy and vascular complications, G-CSF stimulation resulted in a decrease in PB SP levels (Fig. 7d, *p* < 0.01 vs non-diabetic individuals, *p* < 0.05 vs diabetes without complications). We also found an association between changes in SP concentrations and the degree of CD34^+^ HSPC mobilisation in response to G-CSF. In fact, CD34^+^ HSPC mobilisation was significantly higher in individuals showing an increase in SP concentrations compared with those showing unchanged or decreased SP levels (Fig. 7e, *p* < 0.05).

## Discussion

The results from this study represent a significant step forward in the understanding of HSPC pathobiology and its association with nociceptive dysfunction in patients with diabetes. Although an alteration of sympathetic innervation has been documented previously and suggested to be the cause of diabetic mobilopathy [20, 25, 26], this is the first demonstration of the degeneration of specific nociceptive fibres in BM.

We previously demonstrated that tissue ischaemia triggers a neuronal mechanism involving peripheral and central nociceptors, leading to generation of an SP gradient that favours the release of regenerative HSPCs from the BM. HSPCs are recognised as contributing to post-ischaemic recovery through paracrine promotion of reparative vascularisation and inhibition of apoptosis [27]. Extending this concept, here we show for the first time that diabetes remarkably blunts both the SP gradient and NK1R-HSPC release upon induction of LI. The subpopulation of nociceptive-sensitive NK1R-HSPCs is essential in reparative processes as highlighted by studies in mice with BM reconstitution by *Nk1r*-knockout cells [4]. Importantly, the failure of NK1R-HSPCs to home to the ischaemic limb muscle of diabetic mice translates to poor angiogenesis and delayed blood-flow recovery. The newly described nociceptive alteration is complementary to defects in resident vascular cells and endothelial progenitor cells, all well acknowledged in animals and patients with diabetes.

Bone pain and back pain are the most common side effects of G-CSF; here, we report for the first time that both activation of pain and increased SP following G-CSF stimulation predict the extent of HSPC mobilisation. Importantly, poor mobilisers show a total lack of SP upregulation in their PB. Altogether, these data lend strong support to the concept that nociception plays a role in the regulation of HSPC mobilisation for vascular repair, whereas insensitivity to pain may jeopardise cellular healing mechanisms in diabetes. Prevention and treatment of neuropathy may become an integral component of new strategies to enhance the mobilisation of HSPCs and halt vascular damage, as we and others have shown [28, 29].

### Conclusion and clinical perspectives

Impaired nociception is a typical feature of diabetic neuropathy, which is one of the most common and disabling diabetic complications, affecting millions of people worldwide. We herein provide evidence for the existence of a nociceptive neuropathy in the BM of diabetic patients and animal models of diabetes and provide a comprehensive overview of neurological alterations in the diabetic BM. The translational nature of our work strongly suggests that in both humans and in mice, neural control of BM is affected by diabetes. The notion that sensory neuropathy affects the BM and prevents release of reparative cells identifies a hitherto neglected link between diabetic neuropathy and vasculopathy. Pursuing ways to bypass or overdrive the neurological control of the BM niche is likely to uncover novel therapeutic strategies to counter diabetic vascular disease.

## Electronic supplementary material

ESM Methods (PDF 127 kb)

ESM Table 1 (PDF 84 kb)

ESM Table 2 (PDF 88.6 kb)

ESM Table 3 (PDF 95 kb)

ESM Table 4 (PDF 110 kb)

ESM Table 5 (PDF 114 kb)

ESM Fig. 1 (PDF 262 kb)

ESM Fig. 2 (PDF 101 kb)

ESM Fig. 3 (PDF 127 kb)

ESM Fig. 4 (PDF 330 kb)

## Abbreviations

BM: Bone marrow
CLI: Critical LI
G-CSF: Granulocyte colony-stimulating factor
HSPC: Haematopoietic stem and progenitor cell
LI: Limb ischaemia
NK1R: Neurokinin 1 receptor
PB: Peripheral blood
SP: Substance P
T2DM-N: Type 2 diabetes mellitus with peripheral neuropathy
T2DM-NI: Type 2 diabetes mellitus with peripheral neuropathy and CLI
T2DM-U: Uncomplicated type 2 diabetes mellitus
