# Supplementary material for: Sensory neuropathy hampers nociception-mediated bone marrow stem cell release in mice and patients with diabetes
Source: Diabetologia. 2015 Sep 10;58(11):2653–62. doi: 10.1007/s00125-015-3735-0 (PMC4589553; doi:10.1007/s00125-015-3735-0)
Supplement: Supplementary file 1 — (PDF 127 kb) [file 125_2015_3735_MOESM1_ESM.pdf]

ELECTRONIC SUPPLEMENTARY MATERIAL

Detailed Methods

Human studies:

First study Inclusion/exclusion criteria and references for clinical data collection. Only T2DM patients diagnosed from more than 5 years according to American Diabetes Association (ADA) criteria were included. Exclusion Criteria were: age <18 or >80 years, body mass index >36 kg/m^2, pregnancy, dialysis, severe heart failure (IV class New York Heart Association), recent (within 6 months) myocardial infarction or ictus, cancer with adverse prognosis or chemotherapy, steroid-associated immunosuppression, organ transplantation, recent trauma or surgery, and lack of consent to participate to the study.

Diabetic retinopathy was identified based on the patient's personal record of previous retinal fundus examination. Clinical nephropathy was represented by spot microalbuminuria or 24-hour macroalbuminuria or eGFR <60 ml/min/1.73 mq (calculated using the Modification of Diet in Renal Disease formula) [1, 2]. The following data were collected in type 2 diabetic patients and control subjects: age, sex, height, weight, waist circumference, HbA_{1c}, DM duration, history of smoke, hypertension, dyslipidemia and cardiovascular disease. We also collected information on diabetic complications as described above.

Patient-level trial data were confidentially provided by Roche Pharmaceuticals on the basis of a non-commercial data transfer agreement. The company had no role in the design and interpretation of the analysis performed for this study.

Animal studies:

Experiments were performed in accordance with the Guide for the Care and Use of Laboratory Animals (The Institute of Laboratory Animal Resources, 1996) and with approval of the University of Bristol and the British Home Office (Licence: 30/2811). As a type 2 diabetic model, we used 16 weeks-old male obese leptin-receptor homozygous mutant BKS.Cg-*Lepr*^{db}/*Lepr*^{db}/OlaHsd (*db/db*) mice (Harlan, UK). Elevation of blood glucose starts at 4-8 weeks of age, followed by manifestation of neuropathy [3]. Age- and sex-matched lean (BKS.Cg-m$^{+/+}$*Lepr*^{db}/OlaHsd) (*db/+*) mice served as controls. Animals were housed with an inverse 12 hours day-night cycle with lights on at 8:30pm in a temperature (22±1ºC) and humidity (55±5%) controlled room. All mice were allowed free access to water and a maintenance normal food. As a model of reparative neovascularization, we applied unilateral limb ischaemia (LI), induced by ligature of the left femoral arteries described previously [4]. Mice were anesthetized with 2,2,2tribromoethanol (0.3gm/kg, i.p), unilateral limb ischaemia (LI) was induced using a refined procedure which consists of ligation with a 7-0 silk suture and electro-coagulation of the left femoral artery. During the postoperative period, pain was relieved by a subcutaneous administration of analgesic. Mice were killed in groups at 0, 3, 7, 14, 21 days after LI (n=5 mice per time point) and peripheral blood (PB) and BM from tibias and femurs were collected for neuropeptide levels assessment and cell antigenic profile analysis.

Flow Cytometry:

Cell isolation: (*i*) Whole blood was collected in tubes containing EDTA at a final concentration of 0.005 mol/l. (*ii*) BM cells were obtained by flushing the femoral marrow of mice. The contralateral side was used in case of mice undergoing LI. In human studies, the marrow was either sampled from the posterior iliac crest using a stainless steel needle or collected from bone remnants of hip surgery. Freshly harvested BM cells were washed with ice-cold Hank balanced salt solution containing 0.5% bovine serum albumin and

0.02% sodium azide. (*iii*) SCs were also isolated from adductor muscles of mice undergoing LI. Muscles were harvested, minced with fine scissors and placed into a digest solution of Collagenase A 100 µg/µl (Roche), Dispase II (1X) 2.4 U/ml (Roche), DNase I 10 mg/ml (Roche), BSA 10%(w/v), $CaCl_2$ 0.05 mol/l, $MgCl_2$ 1 mol/l in PBS at 37°C for 1 h, triturated through 40µm diameter nylon mesh, washed with 0.2% BSA + 0.1% DNAse I in PBS.

Analyses: (*i*) Mouse cells were fixed and permeabilized with cytofix/cytoperm solution (BD), blocked with Fc Block (Fc, eBioscience anti mouse CD16/32) and stained with antibodies against the following markers: Lineage Mixture (mouse CD3e, CD11b, CD45R, Ly-6C/G, TER119, Caltag), Ly6-A/E (Sca-1), Cd117 (c-Kit), and NK1R (immunostar). The final concentration of primary antibodies is reported in (**ESM Tab. 5**). Cells were then washed and treated with Goat Anti-Rabbit PE secondary antibodies. (*ii*) Human cells were labeled with: anti-NK1R FITC rabbit polyclonal (Novus Biologicals), anti-CD34 PE-Cy7 or APC-conjugated as indicated (both BD biosciences) and anti-KDR PE mouse monoclonal (R&D system). Analyses of antigenically defined cell populations were performed using a FACS Canto II equipped with FACS Diva software (BD Biosciences).

Immunohistochemistry and immunofluorescence:

Decalcified human BM biopsy or femoral BM were embedded in paraffin, sectioned on a rotary microtome at 2µm, and then dried, deparaffinized and rehydrated. Enzymatic epitope retrieval was performed by microwave, boiled in sodium citrate buffer pH6. (*ii*) Mouse femoral bones were collected and cleaned from surrounding tissues, fixed with 4% paraformaldehyde for 24 hours at 4˚C and decalcified in 10% formic acid for 48 hours at 4˚C. Then, after a brief post-fixation with 4% paraformaldehyde for 4 hours at 4˚C, the bones were rinsed in PBS and processed for paraffin embedding. Four µm-thick histological sections were dried, deparaffinised and rehydrated. The antigen retrieval was performed by microwave, boiled in sodium citrate buffer pH6. (*iii*) In immunohistochemistry protocols using diaminobenzidine (DAB) and alkaline phosphatase (AP), endogenous peroxidase was blocked with H_2O_2 3%. Dako REAL EnVision/HRP, Rabbit/Mouse (ENV) was used for detection of primary antibodies as described in **ESM Tab. 5**. The reactions were revealed by solution (1:50) of Dako REAL DAB+ Chromogen and Dako REAL Substrate Buffer. Nuclei were stained with Mayer's hematoxylin. All staining steps were performed at room temperature. (*iv*) In immunofluorescence protocols, tissue sections were blocked with non-immune normal serum from the species of the secondary antibody (Sigma-Aldrich). Sections were then incubated with primary antibodies as shown in **ESM Tab. 5**. Secondary antibodies were goat anti-rat, anti-rabbit and anti-mouse IgG (Alexafluor) or streptavidin conjugated. The incubation was 60 min at 37°C for human samples and room temperature for mouse samples. This was followed by an amplification step using Fluorescein Tyramide Signal Amplification System (Perkin Elmer). Nuclei were stained with Dapi and slides were mounted using fluorescence mounting medium (Dako) or Prolong Gold (Invitrogen). (*v*) Morphometric analyses of different BM components including nerve fibres and microvessels was performed on images captured with a digital camera at final magnification of 40X, using an image analysis software (Image pro-plus 4.0, Media Cybernetics, USA).

Supplemental References

[1] Molitch ME, DeFronzo RA, Franz MJ, Keane WF, Mogensen CE, Parving HH (2003) Diabetic nephropathy. Diabetes Care 26 Suppl 1: S94-98

[2] Levey AS, Bosch JP, Lewis JB, Greene T, Rogers N, Roth D (1999) A more accurate method to estimate glomerular filtration rate from serum creatinine: a new prediction equation. Modification of Diet in Renal Disease Study Group. Ann Intern Med 130: 461-470

[3] Muller KA, Ryals JM, Feldman EL, Wright DE (2008) Abnormal muscle spindle innervation and large-fiber neuropathy in diabetic mice. Diabetes 57: 1693-1701

[4] Madeddu P, Emanueli C, Spillmann F, et al. (2006) Murine models of myocardial and limb ischemia: diagnostic end-points and relevance to clinical problems. Vascul Pharmacol 45: 281-301
