# Supplementary material for: Sensory neuropathy hampers nociception-mediated bone marrow stem cell release in mice and patients with diabetes
Source: Diabetologia. 2015 Sep 10;58(11):2653–62. doi: 10.1007/s00125-015-3735-0 (PMC4589553; doi:10.1007/s00125-015-3735-0)
Supplement: Supplementary file 2 — (PDF 84 kb) [file 125_2015_3735_MOESM2_ESM.pdf]

ESM Tab. 1: Clinical Study 1, an observational study with the main objective of assessing the association between diabetes mellitus and nerve rarefaction in the bone marrow.

	**Controls (N=49)**	**Diabetics (N=94)**	**p-value**
Age (years)	68.0±11.1	63.8±8.8	0.014
Sex (males) (n,%)*	29 (59)	76 (81)	0.005
Body Mass Index (kg/m^2)	26.4±3.0	30.4±5.5	<0.001
Fasting glucose (mmol/l)	4.9±05	8.4±2.9	<0.001
HbA1c (%)<br>(mmol/mol)	4.9±0.5<br>(30±5.5)	7.8±1.7<br>(62±18.6)	<0.001

Data are presented as mean±SD. *(n,%) number and percentage of males.
