# Supplementary material for: Sensory neuropathy hampers nociception-mediated bone marrow stem cell release in mice and patients with diabetes
Source: Diabetologia. 2015 Sep 10;58(11):2653–62. doi: 10.1007/s00125-015-3735-0 (PMC4589553; doi:10.1007/s00125-015-3735-0)
Supplement: Supplementary file 3 — (PDF 88.6 kb) [file 125_2015_3735_MOESM3_ESM.pdf]

ESM Tab. 2. Clinical Study 1, details of type 2 diabetics subgroups

	T2DM-N (N=39)	T2DM-NI (N=39)	T2DM-U (N=16)	p-value
Age (years)*	60.4 ± 7.9	68.3 ± 7.1	61.1± 10.0	<0.001
Gender (males)*	30(77)	34(87)	12(75)	0.417
Smoking (n, %)**	0(0)	1(3)	1(6)	0.335
Hypertension (n, %)**	35(90)	39(100)	4(25)	<0.001
Body Mass Index (kg/m^2)*	30.6 ± 5.8	29.9 ± 5.1	30.7 ± 5.9	0.837
Duration of diabetes (years)*	12.3±12.5	15.9±9.9	7.4±9.5	0.009
**Laboratory test***				
HbA_{1c} (%)<br>(mmol/mol)	8.1±1.8<br>(65±19.7)	7.9±1.7<br>(63±18.6)	6.8±1.2<br>(51±13.1)	0.039
Fasting glucose (mmol/l)	8.7±2.8	8.2±2.9	7.9±3.2	0.485
Creatinine (µmol/l)	96.8±61.6	114.4±44	70.4±17.6	<0.001
Total cholesterol (mmol/l)	4.45±1.13	4.29± 1.07	4.3±1.05	0.794
LDL-cholesterol (mmol/l)	2.45±0.92	2.39±1.03	2.67±0.81	0.707
HDL-cholesterol (mmol/l)	1.44±0.56	1.34±0.5	1.27±0.57	0.541
Triglycerides (mmol/l)	1.28±0.51	1.63± 0.72	1.31± 0.71	0.052
**Medications****				
Insulin	14(36)	24(62)	1(6)	0.001
Oral anti-diabetic drugs	4(10)	1(3)	12(75)	<0.001
Incretins	4(10)	0(0)	0(0)	0.053
Statins	19(49)	27(69)	8(50)	0.150
Anti-platelet therapy	17(44)	39(100)	6(38)	<0.001

**Complications****				
Retinopathy	19(53)	29(83)	0(0)	<0.001
Non recent MI/ictus	13(33)	29(76)	1(6)	<0.001
Nephropathy	20(54)	37(95)	0(0)	<0.001

Distribution of clinical variables, laboratory tests, therapies and complications in type 2 diabetic patients affected by peripheral neuropathy (T2DM-N), or peripheral neuropathy and CLI (T2DM-NI) and uncomplicated patients (T2DM-U) enrolled in Study 1.

*Data presented as mean±SD; **Data presented as number and percentage.
