# Supplementary material for: Sensory neuropathy hampers nociception-mediated bone marrow stem cell release in mice and patients with diabetes
Source: Diabetologia. 2015 Sep 10;58(11):2653–62. doi: 10.1007/s00125-015-3735-0 (PMC4589553; doi:10.1007/s00125-015-3735-0)
Supplement: Supplementary file 4 — (PDF 95 kb) [file 125_2015_3735_MOESM4_ESM.pdf]

ESM Tab. 3: **Study 2 assessing the expression of the SP receptor NK1R on circulating CD34$^+$ progenitor cells**

**Parameter**	**Non diabetic controls**	**Diabetic patients**	**p-value**
Number	24	74	-
Age, years	64.3±8.8	62.7±9.8	0.494
Sex male, %	47.8	65.7	0.126
BMI, kg/m2	26.0±3.7	28.0±4.0	0.030
HbA_{1c}, % (mmol/mol)	5.4±0.4 (36±4.4)	7.2±1.1 (55±12.0)	<0.001
Diabetes duration, years	-	9.0±8.0	-
Hypertension, %	34.8	50.7	0.186
Smoking, %	10.0	13.2	0.704
**Complications**			
Diabetic retinopathy, %	-	25.3	-
Albuminuria, mg/g	9.2±2.8	34.0±70.1	0.096
Diabetic neuropathy, %	-	19.2	-
Macrovascular disease, %	0.0	56.7	<0.001
**Medications**			

Insulin, %	-	30.1	-
Incretins, %	-	16.4	-
Oral anti-diabetic drugs, %	-	67.5	-
Statins, %	30.4	75.3	<0.001
ACE inhibitors, %	34.8	72.6	0.001
Other anti-hypertensive drugs, %	13.0	36.9	0.031
Anti-platelet therapy, %	13.0	39.7	0.017

Distribution of clinical variables, laboratory tests, complications and therapies in non diabetic controls and type 2 diabetic patients enrolled in Study 2.
