# Supplementary material for: Sensory neuropathy hampers nociception-mediated bone marrow stem cell release in mice and patients with diabetes
Source: Diabetologia. 2015 Sep 10;58(11):2653–62. doi: 10.1007/s00125-015-3735-0 (PMC4589553; doi:10.1007/s00125-015-3735-0)
Supplement: Supplementary file 5 — (PDF 110 kb) [file 125_2015_3735_MOESM5_ESM.pdf]

ESM Tab. 4: **Study 4 assessing the CD34[+] cell mobilization response to low-dose human recombinant G-CSF**

Parameter	All patients			Non diabetic patients			Diabetic patients		
	No pain	Pain	p	No pain	Pain	p	No pain	Pain	p
Diabetes, %	71.9	54.5	0.301	0.0	0.0	-	100.0	100.0	-
Age, years	48.0±8.5	39.5±11.9	0.071	45.8±15.3	30.0±13.4	0.042	48.9±10.2	47.3±19.3	0.794
Sex male, %	84.0	73.0	0.404	89.0	60.0	0.238	83.0	83.0	0.968
BMI, kg/m2	28.0±5.0	26.3±7.9	0.451	26.7±9.5	24.2±10.8	0.359	28.4±5.9	28.1±11.5	0.914
HbA_{1c}, % (mmol/mol)	8.1±1.7 (65±18.6)	7.5±3.0 (58±32.8)	0.271	-	-	-	8.1±1.7 (65±18.6)	7.5±3.0 (58±32.8)	0.271
Diabetes duration, years	15.9±3.3	13.8±5.7	0.674	-	-	-	15.9±3.3	13.8±5.7	0.674
Hypertension, %	53.0	27.0	0.145	22.0	0.0	0.290	65.0	50.0	0.511
**Complications**									
Diabetic retinopathy, %	18.8	18.2	0.967	-	-	-	26.0	33.3	0.734
Microalbuminuria, %	9.4	0.0	0.303	0.0	0.0	-	13.0	0.0	0.368
Diabetic neuropathy, %	21.9	9.1	0.359	-	-		30.4	16.7	0.518
Macrovascular disease, %	25.0	9.0	0.274	0.0	0.0	-	34.7	16.7	0.411

**Medications**									
Insulin, %	59.0	45.0	0.435	-	-	-	82.6	83.3	0.968
Incretin, %	9.0	9.0	1.000	-	-	-	13.0	16.7	0.826
Oral antidiabetic agent, %	38.0	18.0	0.248	-	-	-	52.2	33.3	
Statin, %	38.0	27.0	0.550	0.0	0.0	-	52.0	50.0	0.928
ACE inhibitors, %	44.0	18.0	0.136	11.0	0.0	0.478	57.0	33.0	0.329
Other Anti-hypertensive drug, %	25.0	18.0	0.654	11.0	0.0	0.478	35.0	33.0	0.949
Anti-platelet therapy, %	25.0	18.0	0.654	0.0	0.0	-	35.0	33.0	0.949

Clinical characteristics, complications and therapy of DM patients in the clinical study 4. Data expressed as mean±SD.
