# Supplementary material for: Sensory neuropathy hampers nociception-mediated bone marrow stem cell release in mice and patients with diabetes
Source: Diabetologia. 2015 Sep 10;58(11):2653–62. doi: 10.1007/s00125-015-3735-0 (PMC4589553; doi:10.1007/s00125-015-3735-0)
Supplement: Supplementary file 6 — (PDF 114 kb) [file 125_2015_3735_MOESM6_ESM.pdf]

ESM Tab. 5: Antibodies used for immunostaining and flow cytometry.

Antibody	Manufacturer	Code	IHC dilution	FACS dilution	Detection system	IHC dilution	FACS dilution
Mouse Anti-PGP9.5 [31A3]	Abcam	ab20559	1:500		Peroxidase/DAB and System AP	-	
Rat Anti-Substance P [M09205]	Abcam	ab7340	1:100		Alexa Fluor 555	1:200	
Rabbit Anti-Substance P	Novus Biologicals	NBP1-78326	1:200		Peroxidase/DAB	-	
Mouse Anti-Tyrosine Hydroxylase [779427]	R&D systems	MAB7566	1:20		Peroxidase/DAB	-	
Mouse Anti-Human CD68 [PG-M1]	Dako	M0876	1:100		Peroxidase/DAB	-	
Mouse Anti-Human CD34 [QBEnd10]	Dako	M7165	1:50		Alexa Fluor 555	1:20	
Rabbit Anti-NK1 receptor	Novus Biologicals	NB300-119B	1:50		Streptavidin Alexa Fluor 488	1:200	
Rabbit Anti-NK1 receptor - FITC	Novus Biologicals	NB300-119G		1:30			
Mouse Anti-Human CD34 - PE-Cy7 [MOPC-21]	BD Biosciences	348811		1:20			
Mouse Anti-Human KDR - PE [89106]	R&D Systems	FAB357P		1:20			
Rabbit Anti-PGP9.5	Millipore	AB5925	1:500		Peroxidase/DAB or TSA plus Fluorescein kit(NEL741E001KT)	1:50	
Rabbit Anti-Substance P	Immunostar	22064	1:2500		Peroxidase/DAB or TSA plus Fluorescein kit(NEL741E001KT)	1:50	

Rabbit Anti-NGF*	Abcam	Ab6199	1:500		Peroxidase/DAB	-	
Rabbit Anti-NK1	Immunostar	20060	1:50	1:500	Goat anti rabbit PE	1:300	
Rat Anti-mouse CD31 [MEC13.3]	BD Pharmingen	557355	1:100		Gaot anti rat Alexa Fluor 568	1:300	
Rat Anti-CD117 APC-eFluor 780	BD Biosciences	47-1171-82		1:400			
Rat Anti-Ly6-A/E(Sca-1) PerCP-Cy5.5	BD Biosciences	45-5981-80		1:400			
Mouse Anti- CD3e, CD11b, CD45R,Ly-6C/G, TER119 (Lineage cocktail) Alexa Fluor 488	Invitrogen	MLM20		1:00			
Isolectin B4 - Biotin	Invitrogen	121414	1:100		Streptavidin Alexa Fluor 568	1:200	
