# Supplementary material for: Sensory neuropathy hampers nociception-mediated bone marrow stem cell release in mice and patients with diabetes
Source: Diabetologia. 2015 Sep 10;58(11):2653–62. doi: 10.1007/s00125-015-3735-0 (PMC4589553; doi:10.1007/s00125-015-3735-0)
Supplement: Supplementary file 7 — (PDF 262 kb) [file 125_2015_3735_MOESM7_ESM.pdf]

ESM Fig. 1

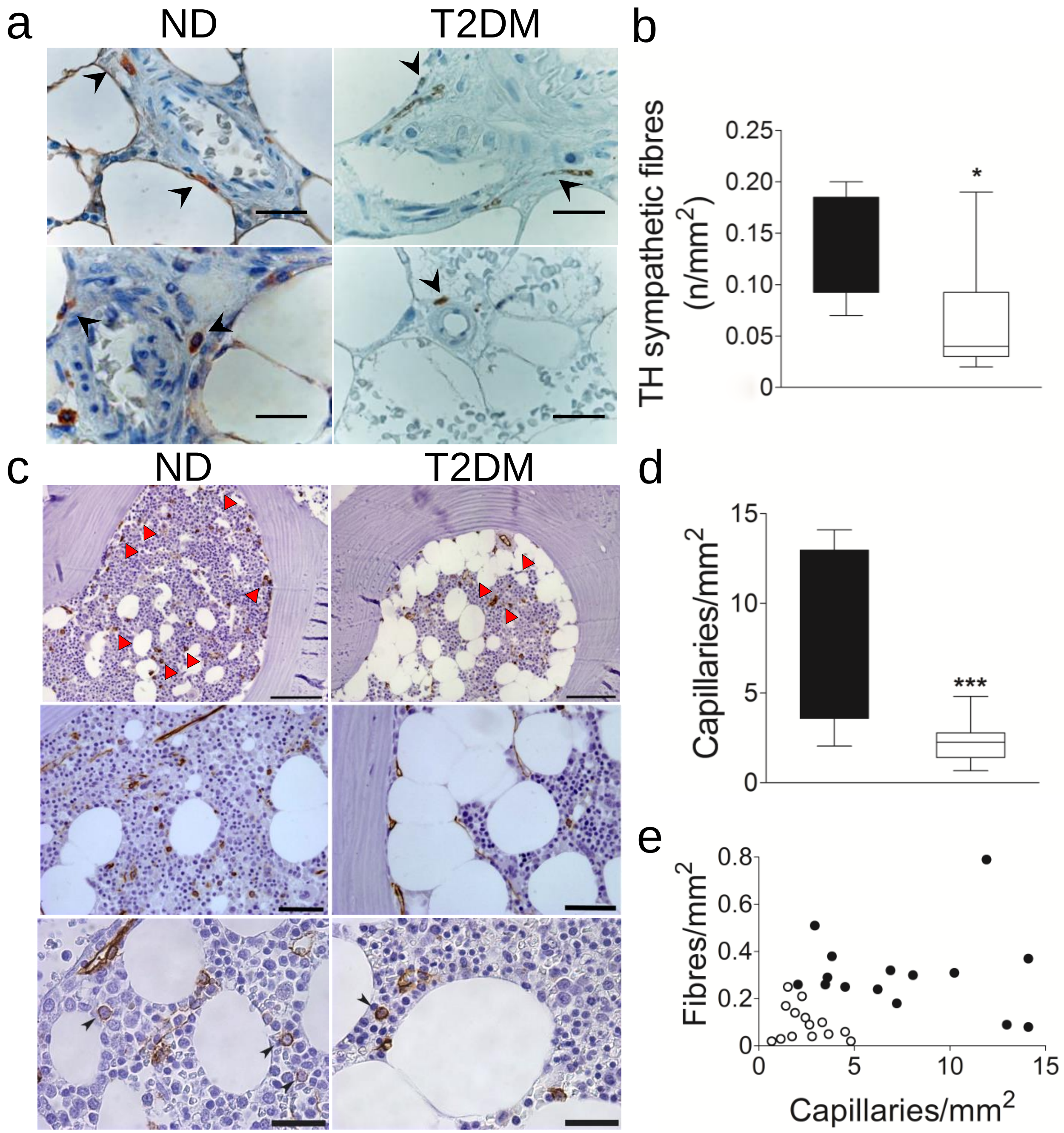

ESM Figure 1: Association of sensory neuropathy and microangiopathy in BM of diabetic patients. (**a**) Nerve fibre rarefaction in BM of T2DM was confirmed by staining for the sympathetic nerve marker tyrosine hydroxylase (TH). Representative microphotographs (scale bar 20µm) and box and whiskers graphs (**b**) illustrating the difference between ND (black, n=6) and diabetic subjects (white, n=8). *P<0.05 vs. ND. (**c**) Immunohistochemistry of BM microvasculature using a CD34 antibody. Typical vascular structures are pointed by red arrowheads. Abundance of adipose tissue in diabetic BM and reduction of the hematopoietic tissue is associated with microvascular rarefaction, with vessel being mainly confined in the central part of the marrow (scale bar top pictures 100µm and mid pictures 50µm). High magnifications in the lower photo (scale bar 30µm) showing CD34 positive hematopoietic cells, pointed by black arrowheads. (**d**) Box and whiskers graphs showing the reduced capillary density in BM of type diabetic (white) compared with ND (black) (***P<0.001) and (**e**) plot graph showing the correlation between capillary and nerve fibre density in BM of ND (blacks dots, n=15) and type 2 diabetic subjects (white dots, n=14).
