# Supplementary material for: Sensory neuropathy hampers nociception-mediated bone marrow stem cell release in mice and patients with diabetes
Source: Diabetologia. 2015 Sep 10;58(11):2653–62. doi: 10.1007/s00125-015-3735-0 (PMC4589553; doi:10.1007/s00125-015-3735-0)
Supplement: Supplementary file 8 — (PDF 101 kb) [file 125_2015_3735_MOESM8_ESM.pdf]

**ESM Fig. 2**

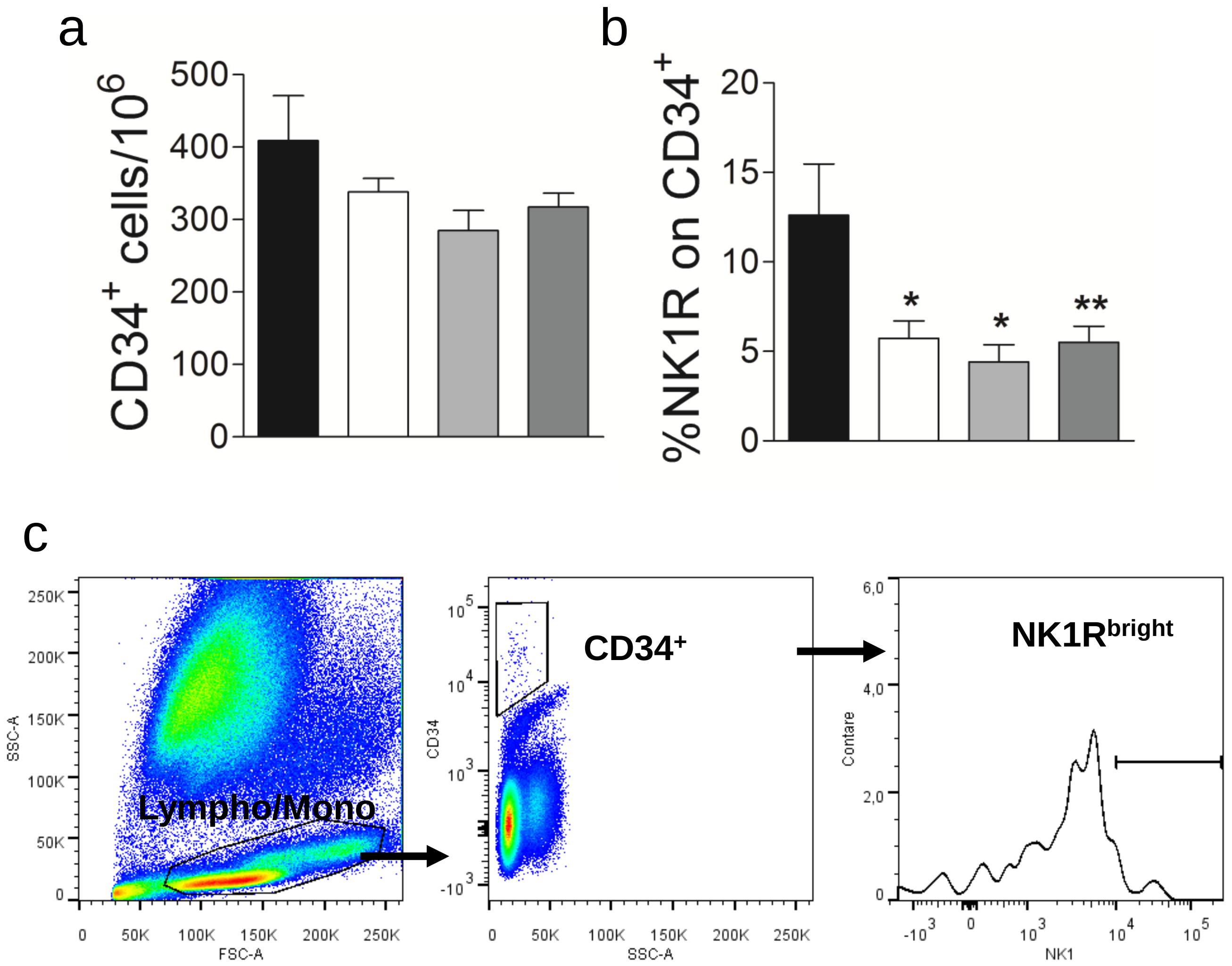


**ESM Figure 2: Reduced NK1R expression in stem cells of diabetic patients**. $CD34^{+}$ cell count (**a**), and the respective expression of the SP receptor NK1R (**b**) were quantified in non-diabetic (black) and type 2 diabetic patients divided according to complications (none, white; neuropathic, light grey; or neuroischaemic, dark grey, *$P$<0.05 vs. ND. Panel **c** shows a representative FACS plot to illustrate the gating strategy used to assess NK1R expression on $CD34^{+}$ cells.
