# Supplementary material for: Sensory neuropathy hampers nociception-mediated bone marrow stem cell release in mice and patients with diabetes
Source: Diabetologia. 2015 Sep 10;58(11):2653–62. doi: 10.1007/s00125-015-3735-0 (PMC4589553; doi:10.1007/s00125-015-3735-0)
Supplement: Supplementary file 9 — (PDF 127 kb) [file 125_2015_3735_MOESM9_ESM.pdf]

ESM Fig. 3

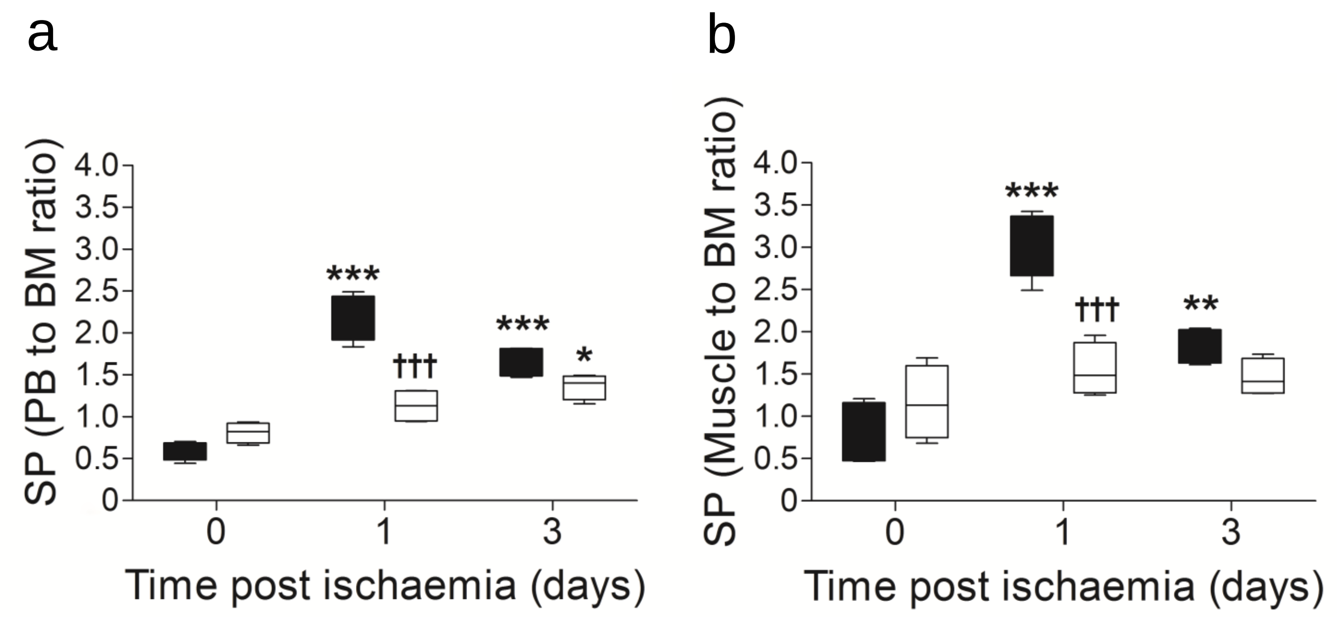

ESM Figure 3: Diabetes alters the formation of a gradient of SP following ischaemia. Bar graphs show the SP ratio between PB and BM (**a**) and ischaemic adductor muscle and BM (**b**). Black: non-diabetic, white: type 2 diabetic mice. **P*<0.05, ***P*<0.01 and ****P*<0.001 vs. time 0**;** †††P<0.001 vs. ND, n=5 per group.
