# Supplementary material for: Sensory neuropathy hampers nociception-mediated bone marrow stem cell release in mice and patients with diabetes
Source: Diabetologia. 2015 Sep 10;58(11):2653–62. doi: 10.1007/s00125-015-3735-0 (PMC4589553; doi:10.1007/s00125-015-3735-0)
Supplement: Supplementary file 10 — (PDF 330 kb) [file 125_2015_3735_MOESM10_ESM.pdf]

ESM Fig. 4

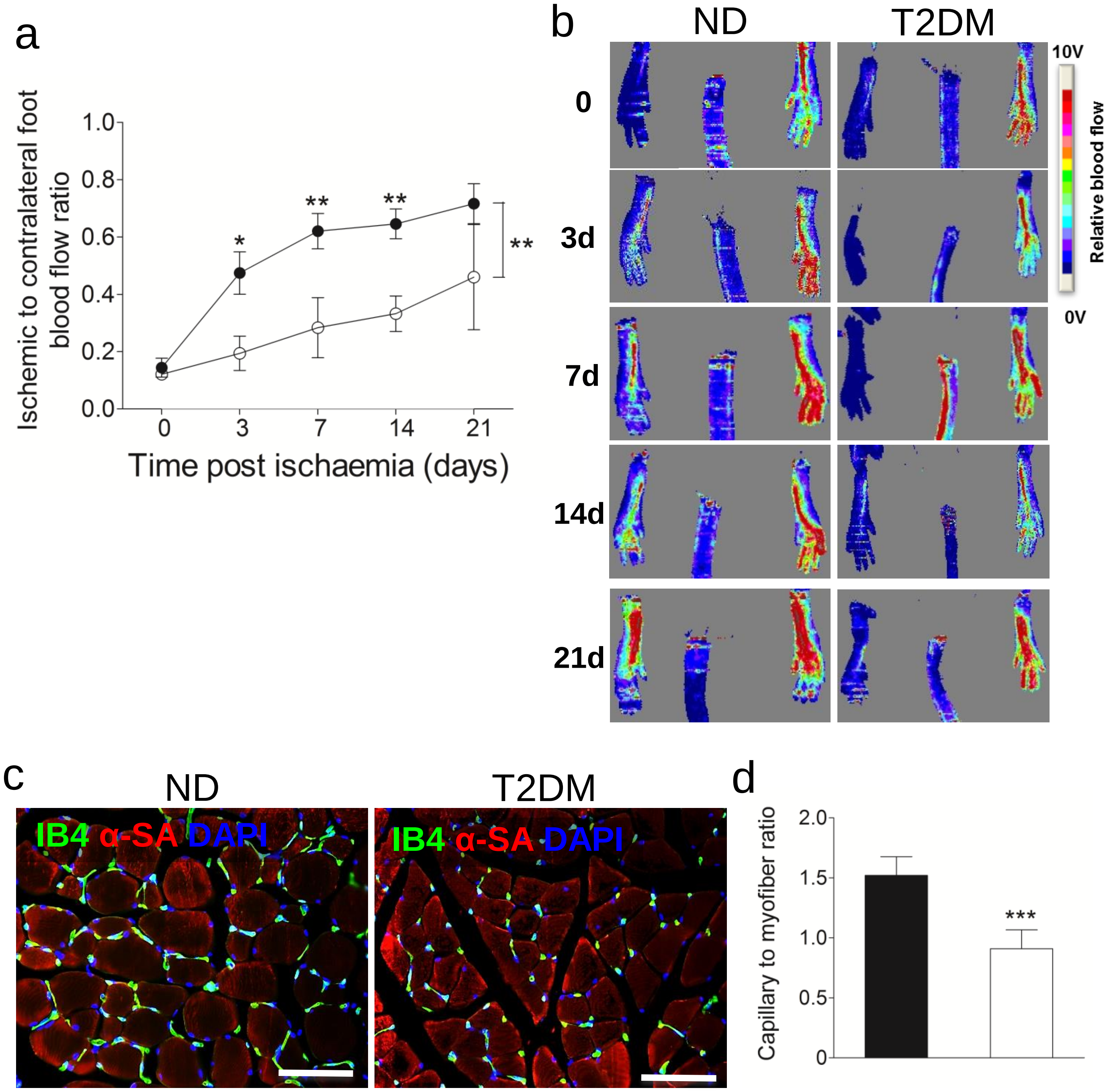

ESM Figure 4: : Impaired nociceptive mechanism is associated with delayed perfusion recovery, impaired angiogenesis in type 2 diabetic mice submitted to unilateral limb ischaemia. (**a**) Line graph show the reduced blood flow recovery of type 2 diabetic mice following induction of limb ischaemia. Black: non-diabetic, white: type 2 diabetic mice. (**b**) Representative images of blood flow assessed by laser Doppler flowmetry. (**c**) Representative images of capillaries in ischemic muscles. Isolectin B4 (IB4), α-Sarcomeric Actin (α-SA) (scale bar 50μm). (**d**) Reduced capillary density (means and SE) in ischemic muscles of type 2 diabetic mice. Black: non-diabetic, white: type 2 diabetic mice. *P<0.05, **P<0.01 and ***P<0.001 vs. ND, n=5 per group.
